# Improved disease diagnosis system for COVID-19 with data refactoring and handling methods

**DOI:** 10.3389/fpsyg.2022.951027

**Published:** 2022-08-12

**Authors:** Ritesh Jha, Vandana Bhattacharjee, Abhijit Mustafi, Sudip Kumar Sahana

**Affiliations:** Department of Computer Science and Engineering, Birla Institute of Technology, Mesra, Ranchi, India

**Keywords:** classification, disease diagnosis, COVID-19, data augmentation, data pre-processing

## Abstract

The novel coronavirus illness (COVID-19) outbreak, which began in a seafood market in Wuhan, Hubei Province, China, in mid-December 2019, has spread to almost all countries, territories, and places throughout the world. And since the fault in diagnosis of a disease causes a psychological impact, this was very much visible in the spread of COVID-19. This research aims to address this issue by providing a better solution for diagnosis of the COVID-19 disease. The paper also addresses a very important issue of having less data for disease prediction models by elaborating on data handling techniques. Thus, special focus has been given on data processing and handling, with an aim to develop an improved machine learning model for diagnosis of COVID-19. Random Forest (RF), Decision tree (DT), K-Nearest Neighbor (KNN), Logistic Regression (LR), Support vector machine, and Deep Neural network (DNN) models are developed using the Hospital Israelita Albert Einstein (in São Paulo, Brazil) dataset to diagnose COVID-19. The dataset is pre-processed and distributed DT is applied to rank the features. Data augmentation has been applied to generate datasets for improving classification accuracy. The DNN model dominates overall techniques giving the highest accuracy of 96.99%, recall of 96.98%, and precision of 96.94%, which is better than or comparable to other research work. All the algorithms are implemented in a distributed environment on the Spark platform.

## Introduction

COVID-19 is a term that most people on this planet have got accustomed to and almost all families have been psychologically physically affected by this. The “CO” stands for corona, the “VI” for virus, and the “D” for the disease. It was previously known as “2019 novel coronavirus” or “2019-nCoV.” The first patient or “patient-zero” of this deadly virus was probably a 55-year-old individual from Hubei province in China, and this incident dates back to November 17, 2019. A month later, several cases of people infected with the virus started being reported from Wuhan, in Hubei province. It has been declared a pandemic by World Health Organization (WHO) due to widespread infection. Several countries went on months of lockdown, while others decided to quarantine the infected individuals. Over a million people have died all over the world due to COVID-19 (National Institute of Infection Diseases, 2020; World Health Organization, 2020a,b). Since the first few cases were reported, researchers around the world have been trying to find a way to contain the spread of this virus. But upon studying various COVID positive cases, it has been concluded that the infection rate and the recovery rate could not be calculated easily, as several factors came into the picture. Some of them are days since first coming into contact with the virus, days since first showing symptoms, previous history of respiratory diseases or conditions, any other underlying disease or ailment, etc. With further studies and research, it has been possible to utilize many latest technologies like big data, machine learning, artificial intelligence, etc. in working toward the prevention of the further spread of the virus.

In this work, keeping in view the fact that real-world data analysis deals with large and complex data with high dimensionality and volume, and require some sort of pre-processing, our focus has been in giving a comprehensive methodology for data handling. This would enable the future researchers in conducting their studies. We note that pre-processing or transformation of this data impacts the accuracy of the prediction this data is used for, and hence is an important consideration. The objective of this research paper is to prepare the data in a manner that would be suitable as input for downstream machine learning task of classifying COVID-19 patients. For this, data pre-processing, feature selection, and data augmentation have been applied. This results in creation of five datasets, namely DR1, DS1, DS2, DS3, and DS4. The K-Nearest neighbor (KNN), Decision trees (DTs), RF, and Neural network classifiers were applied on all the datasets for prediction of COVID-19 disease. Results obtained have been compared with other research work.

The main contributions of this research work are:

• Addresses an important issue of lack of sufficient data for disease prediction models by elaborating on data handling techniques.
• Feature selection techniques have been used to select the right features so as to build an efficient classifier.
• Computations have been done in a distributed environment so as to build real time models when necessary.

The rest of this paper is organized as follows: Section “Related Work” presents the literature review. Section “Data handling” presents data handling explaining the data preparation and the evaluation parameters. Section “Methods and experimental setup” presents the description of the methods and experimental setup. Section “Experiments and results” presents the experiments and results, and finally, section “Conclusion” presents the conclusion.

## Related work

Researchers have extensively conducted studies related to COVID-19 and made interesting observations. One such research is made by Fan et al. (2020) who have performed an observational study that attempts to decipher the role of occupation in the spread of COVID-19. Steven et al. (2020) studied that the reproductive value of the virus is somewhere between 4.7 and 6.6, which is much higher than the original estimate. Zhao et al. (2020) attempted to find the number of unreported cases in the first half of January 2020 in China using maximum likelihood criteria. Salazar et al. (2020) have tried to find out the places that may have unreported imported cases based on international air travel estimates from Wuhan to other parts of the world. Researchers in the clinical domain as well as technology domain have been interested in applying advance technologies for improving healthcare. Artificial intelligence and Machine learning techniques have also played a major role (Jiang et al., 2017). Jianguo et al. (2018) have proposed a DDTRS (Disease Diagnosis and Treatment Recommendation System), maximizing the utilization of advanced healthcare technology and knowledge of experienced doctors using clustering and association rule mining techniques. With the increasing inclusion of technology in healthcare, medical data in the form of images, keeps growing day by day. Its volume has gone up to such an extent that now it is impossible for traditional methods to handle it. With a volume so huge, healthcare data is best suited to be handled by big data technology, as presented by Kouanou et al. (2018). A detailed review of data mining techniques using big data for healthcare is presented by Herland et al. (2014). Asthma related emergency department visits have been studied using big data (Ram et al., 2015).

Presented now are some of the recent research works conducted related to COVID-19 pandemic. Ranjeet Singh has conducted research using regression analysis to examine the COVID-related data for India (Singh, 2020). The biggest challenge COVID has brought upon developing countries is that of diagnosing an infected person with the virus, with a very less number of diagnosis kits to be spared. Authors in Batista et al. (2020) have attempted to resolve this issue by predicting the risk involved in positive COVID diagnosis using machine learning, with results only from urgent care admission tests as predictors. Mondal et al. (2020) discuss the probable applications of data analytics for the COVID-19 disease diagnosis and obtain the best results with Multi-Layer Perceptrons at 93.13% accuracy. Zobai et al. (2021) predicted the risk of positive COVID-19 diagnosis using the emergency care admission exams, using machine learning classifiers.

Jiang et al. (2020) developed predictive models to identify features which would play an important role. Schwab et al. (2020) applied XGBoost on COVID-19 dataset and achieved an AUC (Area under curve) value of 0.66. BurakAlakus and Turkoglu (2020) presented a comparison of deep learning approaches in COVID-19 prediction. Other researchers have studied the impact of this disease on hospitalized patients (Wang et al., 2020), on children (Jiehao et al., 2020; Karm et al., 2020), and other scenarios (Fang et al., 2020; Huang et al., 2020; Jamshidi, 2020; Li et al., 2020; Wynants et al., 2020; Wölfel et al., 2020). Abdulkareem et al. (2021) applied machine learning techniques and IoT in smart hospital environment and achieved an accuracy of 95%. Turabieh et al. (2021) obtained the accuracy of 80% applying the CNN model. Parameters of full-blown blood counts have been analyzed by Banerjee et al. (2020) for early detection of the disease and an accuracy of 90% was achieved. Application of XGBoost and CNN by other researchers have given accuracies of 92.67 and 92.52%, respectively (Adem and Kilicarslan, 2021; Podder et al., 2021). Sumayh et al. (2021) provide a prediction method for the early identification of COVID-19 patient’s outcome based on patients’ characteristics monitored at home, while in quarantine. They applied three classification algorithms, namely, logistic regression (LR), random forest (RF), and extreme gradient boosting (XGB) and results showed that RF outperformed the other classifiers with an accuracy of 0.95. In Yuheng (2021) the author qualitatively evaluates the impact of COVID-19 on various biometric systems and to quantitatively evaluate face detection and recognition. The experimental results show that a real-world masked face dataset is essential to build an effective face recognition-based biometric system. Authors in Zheng et al. (2021) propose an automatic segmentation of COVID-19 infected areas using chest computed tomography (CT) scans is critical for assessing disease progression and achieve very good results. Authors in Zhu et al. (2021) conclude that the results from CRP and chest CT scans were indicators of the severity of COVID-19. Wang and Wang (2021) apply edge computing to optimize online education, and a task offloading algorithm is designed to minimize the computing delay of terminal tasks. They propose the construction of education infrastructure, the adjustment of teaching organization, and the learning methods of teachers and students, for enhancement of online education in the COVID-19 pandemic. In their study, Saladino et al. (2020) discuss the issues with mental health caused by the Covid-19 condition. In order to lessen the negative impacts, telepsychology and technology advancements become crucial. The authors demonstrate, using actual data, the value of technological intervention in managing mental stress conditions while saving money because it can be completed online. Khatatbeh et al. (2021) present their study that COVID-19 has impacted the population in Jordan psychologically, specially the females, and the help of professionals would be very much beneficial to overcome such a situation. According to a study by Passavanti et al. (2021) based on data gathered in seven different nations, more than half of the sample has higher-than-average levels of stress, depression, and anxiety as well as PTSD risks. The severity of these disorders is influenced by factors like gender, outdoor activity type, home characteristics, potential contact with infected friends, time spent searching for related information (in the news and social networks), information source type, and, to some extent, income and education level. They came to the conclusion that an individual’s capacity for coping and communication with their environment also had an impact on the impact. In addition to discussing the effects of the coronavirus on these young people’s life, Shukla et al.’s (2021) report on the concerns of Indian teenagers and identify groups of young people who may be particularly prone to negative feelings. Academic success, social and recreational activities, and physical health were the participants’ top concerns. While more men worry about social and leisure activities, more females worry about academic success and physical health. Teenagers in India report that the epidemic has had a substantial influence on a variety of aspects of their lives, and they are especially concerned about their academic performance, social and recreational activities, and physical health. These findings indicate the necessity of ensuring provisions for and access to digital healthcare and education.

## Data handling

### Data preparation

The dataset used has been collected from Hospital Israelita Albert Einstein in São Paulo, Brazil to diagnoses COVID-19. The dataset has 111 features and 5,644 instances. The dataset includes data in anonymous form from the Hospital Israelita Albert Einstein in São Paulo, Brazil, from the patients whose samples were collected to perform the SARS-CoV-2 RT-PCR test during a visit to the hospital (Data4u, 2020). To keep a mean of zero and a standard deviation of one, the hospital made the patient data anonymous, and the clinical data were made standard. This data was made publicly available so that researchers could develop methods to help the hospital in rapidly predicting and potentially identifying SARS-CoV-2 positive patients. Table 1 shows the description and the table listing all the features is given in Supplementary Appendix.

**TABLE 1:** Dataset description.

Name of data set	Positive samples (Class 1)	Negative samples (Class 0)	Number of features
Hospital Israelita Albert Einstein at Sao Paulo, Brazil	558	5,086	111

There is a large imbalance of 558 positive (9.8%) vs. 5,086 negative (91.2%) samples out of a total of 5,644 patients. Preliminary exploratory analysis showed that there were several Null values in each of the feature columns.

A graphical representation shows that there are several null values in the dataset. Different approaches like replacing with zeroes or mean, were adopted to handle this. We used the Feature selection and Data augmentation techniques for dealing with the large number of features and to create a larger dataset for better accuracy.

### Feature selection

Feature selection is the technique for eliminating the less important features and retaining the useful ones for predictive and classification modeling. In our research, we used the DT to obtain the important features of the dataset. The first 44 important features are listed in Table 2.

**TABLE 2:** Features selected after applying distributed decision tree.

Rank	Name of feature	Rank	Name of feature
1	Leukocytes’	23	Patient admitted to intensive care unit
2	Patient age quantile	24	Segmented
3	Red blood Cells	25	Relationship (Patient/Normal)
4	Platelets	26	Strepto A
5	Monocytes	27	Proteina C reativa mg/dL
6	pCO_2_	28	pO_2_ (venous blood gas analysis)
7	Eosinophils	29	Sodium
8	Basophils	30	Hb saturation
9	Mean platelet volume	31	Creatinine
10	Lymphocytes	32	Influenza B
11	Mean corpuscular hemoglobin	33	Influenza A
12	Urea	34	Urine—Leukocytes
13	Rhinovirus/Enterovirus	35	Respiratory Syncytial Virus
14	Adenovirus	36	Urine—Red blood cells
15	Patient admitted to regular ward (1 = yes)	37	pH (venous blood gas analysis)
16	Aspartate transaminase	38	Coronavirus229E
17	Hemoglobin	39	Influenza B
18	CoronavirusNL63	40	Inf A H1N1 2009
19	Red blood cell distribution width (RDW)	41	Coronavirus HKU1
20	Rods #	42	Parainfluenza 3
21	Urine—Aspect	43	Parainfluenza 1
22	Mean corpuscular volume (MCV)	44	Leukocytes

### Generating synthetic data using data augmentation

Let the dataset is defined as *D*(*x*_1_, *x*_2_, ………..*x*_*n*_, *y*), where *x_i_* is known as a feature vector, and y is a vector representing the class label of each instance of the dataset. The data augmentation technique is used to create synthetic data. The function used for this is Gaussian (mean, std), where mean represents the feature vector mean of the dataset and std is the standard deviation of the feature vector of the dataset. Synthetic data is generated using Gaussian (mean, std) + *e* where *e* ∈ *random*(−0.1, 0.1), where function Gaussian is the known as the Gaussian distribution function and *e* is called the noise term.

### Evaluation parameters

The Evaluation Parameters used in this research work are Precision, Recall, F-measure, and Accuracy. Precision (P) measures the ratio of COVID-19 positive predicted patients who are actually positive to the total number of positive class predicted. Recall (R) measures the number of COVID-19 positive predictions made out of all positive patients in the dataset. F-Measure offers a single score that balances both the concerns of precision and recall in one number.


$$F-MeasureFM=2x\frac{Recall\left(R\right)\;xPrecision\left(P\right)}{Recall\left(R\right)+Precision\left(P\right)}$$

Accuracy is the fraction of the COVID-19 positive patients correctly identified.

## Methods and experimental setup

Classification is a two-step process: the first step builds the model based on the training dataset and the class labels available for training. The hypothesis is made and then tested using a test dataset. In the second step, this model is applied for the classification of a new or unlabeled dataset. Neural Network classifier, KNN, DTs, and RF techniques are used in this study. A brief overview of these methods is presented. Then we present our implementation of these algorithms on the Spark distributed platform.

### Deep neural network

Any Artificial Neural Network with several layers between its input and output layers can be classified as a Deep Neural Network (DNN). DNN architectures can generate compositional models with objects in the form of layered composition of primitives, and can model non-linear relationships. Typically, DNNs are feedforward networks with data flowing from the input layer to the output layer without looping back. Deep architectures contain multiple variants of fundamental approaches.

The architecture used in this research has four hidden layers, with 128, 64, 32, 16 nodes in the respective layers. The hidden layer activations functions are ReLu. The output layer has one neuron and Sigmoid activation function.

**FIGURE 1:**
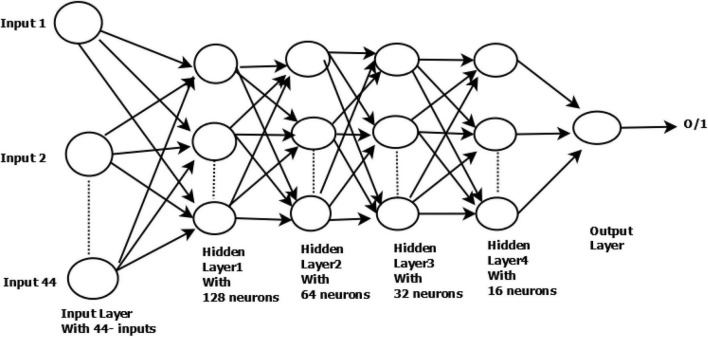
Structure of DNN used in our research work.

In the DNN architecture as shown in Figure 1, the first and last layers are known as the input layer and output layer. Other layers are known as hidden layers. Each layer can have multiple neurons; neurons are known as basic computing units in DNN architecture. The DNN is composed of a backward and feedforward process. The input layer receives the input data and travels from input to output layers in the forward process.

Let $Z_{i}^{h}\left(n\right)$ be the output function of neuron *i* in the layer *h* at epoch *n*, and let $w_{ij}^{h}$be the layer weight between the neuron*i* in the previous layer *h* – 1. The layer *h* – 1 is having *k* neurons.

In the feedforward phase, the layer weights do not change, and the output is evaluated as:


(1) $$Z_{ij}^{h}=\varphi \left(u_{i}^{h}\left(n\right)\right)$$

Where φ is the activation function and is $u_{i}^{h}$ (*n*) defined as:


(2) $$u_{i}^{h}\left(n\right)=\sum_{i=1}^{k}w_{ij}^{h}z_{j}^{h-1}\left(n\right)\;$$

There are various activation functions used in Equation (2). One of the commonly known activation functions is the rectifier linear unit (ReLU). The range of the output stimulus is bounded to [0,1].


(3) φ(ui(n))=min(1,max 0,ui(n)))

In the back-propagation phase, the layer*-l* weights $w_{ij}^{l}\left(n\right)$are adjusted on a layer-by-layer basis using stochastic gradient descent, with a learning rate of 0.0001.

After updating the layer weights in the backward propagation, in each epoch, the forward and backward propagation is executed to reduce the error until the number of epochs is reached.

### Decision tree

DT, is a supervised learning technique and can be used for both classification and regression problems. This classifier is structured like a tree where the internal nodes represent a dataset’s features, the branches represent decision rules, and each leaf node represents the outcomes. A DT represents a graphical form to get all possible solutions to a problem or decision based on given conditions. It is generated by splitting the dataset by choosing important attributes based on attribute selection criteria. In this paper, the “*Gini Index*” is the selection criteria. The other name for *Gini Index*is *Gini impurity*. It gives the probability of a particular data that is incorrectly classified when it is selected randomly. It can be called pure if all elements are linked with a single class. The Gini Index value varies between 0 and 1, with 0 expressing the purity of classification, and 1 indicates that the elements are distributed randomly across various classes. A Gini Index of 0.5 shows that elements are distributed equally over some classes.

### K-nearest neighbor

KNN is one of the simplest machine learning algorithms, which is based on the supervised learning technique. KNN algorithm works by assuming similarities between the new case and K (an integer value) available cases and then putting the new case into the category which is most similar to the available categories. An algorithm’s basic concept is storing all data and then classifies the new data depending on the similarity. Given a training dataset D and test instances *z*, a vector of attribute values, the algorithm computes the distance (or similarity) between *z* and all the training instances to determine its nearest neighbor list. It then assigns a class to *z* by taking the majority of neighboring objects. The *sklearn* library method is used to fit the model, and then it is broadcasted for prediction of testing data in the Spark platform.

### Support vector classifier

It is a supervised machine learning approach that handles both linear and non-linear data to address classification and regression issues. Two or more different classes in a classification problem can be accurately separated by Support vector machine using “hyperplane.” In SVM, the aim is to discover optimal hyperplane separation through training.

### Logistic regression classifier

A statistical analysis method called LR uses previous observations from a data set to predict a binary outcome. By examining the correlation between one or more already present independent variables, a LR model forecasts a dependent data variable.

### Implementation on spark platform

Two algorithms are presented below.

Algorithm 1 describes the KNN implementation in Spark. Step 3 shows the loading of data as Resilient Distributed Dataset. Steps 4 and 5 deal with the null values removal and normalization process. Step 6 presents the use of DTs for finding the important features. Splitting of data into 80% as training and 20% as testing is done in Step 7. Testing data is broadcasted across all the nodes, and this ensures that each node receives exactly same copy of testing data (Step 8). In Step 9, the *sklearn* library *function (fit)* is used for training the model with train data. Once the model has been created, it is broadcasted to each node (Step 10), where it performs the distance computation between training and testing data in each node and finally returns the prediction of the testing data. This is done by predict method in Step 11. All the predictions are collected at the master by the *collect ()* method in Step 12. Finally, accuracy is calculated at the master node (Step 13).

**ALGORITHM 1:** Spark distributed KNN

*1. Input: Set of training data and class label < Tr, y >, number of neighbor < k >, Testing data for prediction < Te > .*
*2. Output: Return class labels of < Te > .*
*3. Data = sc.textFile(file name).*
*4. Pre-processing of data.*
*5. Normalize the data.*
*6. Apply decision tree to obtain important features.*
*7. Split the data in 80% and 20% into Tr, and Ts.*
*8. Ts = sc.broadcast(Ts).*
*9. (i) Model = knn(k).*
*(ii) Model = Model.fit(Tr, y) # y’s are class label of Tr.*
*10. Model = sc.broadcast(Model) # Broadcast the model.*
*11. Rdd = Model.predict(Ts) # predict the class label of broadcasted test instances.*
*12. Classlabel = rdd.collect() # collect all predicted class labels with minimum distances.*
*13. Return accuracy score (True class label testing instances = predicted class label/total no. of testing instance).*

A wide variety of machine learning algorithms have been developed on Spark to deal with big data. We have used the MLib library of Spark to implement DT, RF, and Deep learning for our research. The framework of general algorithms is presented in Algorithm 2.

**ALGORITHM 2:** Classifiers Framework Using MLib

*1. Input: Pre-processed dataset in the form of RDD.*
*2. Output: Accuracy Score.*
*3. From RDD of dataset obtain Dataframe (DF).*
*4. For non-numeric features, apply one-hot encoding.*
*5. Obtain String Indexing of each encoded feature.*
*6. Apply Vector assembler technique for numeric and non-numeric features.*
*7. Divide the data into 80% training and 20% testing data.*
*8. Perform the operation to change the assembled vector into a Pipeline.*
*9. For training, the model applies Spark’s MLLibalgorithms (Decision tree or Random Forest and Deep learning).*
*10. Test the model on testing data to obtain a binary prediction output.*

The experiment has been conducted on a HighPerformanceComputing server (HPC), which has two master nodes and 16 slaves nodes. The master nodes are Intel Xeon E5-2630 v3 2.4 GHz processors with 8 core and 64 GB memory. The CPU compute nodes are two Intel Xeon processor with 8 cores in each processor. The Operating System used was Linux Ubuntu-18.04 with Apache Spark-2.4.3.

## Experiments and results

In our research, the DT is used to obtain the important features of the dataset D1. The first 44 important features as listed in Table 2 are used to create dataset DR1. Next, from D1, we removed the features with more than 99% null values which resulted in a data set with 37 features and 5,644 records. Using this dataset, we applied the data augmentation techniques to generate four different datasets DS1, DS2, DS3, and DS4, the details of which are shown in Table 2. The class label (0) represents the negative class (having no COVID-19) whereas class label (1) represents the positive class (having COVID-19). Missing values were replaced with zeros in all cases.

The details of the datasets generated for our experiments are given in Table 3. Now we present the results of applying the classifiers in Tables 4–8. As seen from Table 4, the highest accuracy of 93.16% and recall of 93.13% are achieved with the DNN classifier applied on dataset DR1. For KNN, we got the best value for *k* = 3. The max_depth for RF is 10, and the number of trees is 100 and we set the max_depth for DT is 10. For DNN, the architecture has four hidden layers, with 128, 64, 32, 16 nodes in the respective layers. The hidden layer activations functions are *ReLu*. The output layer has one neuron and Sigmoid activation function. The *binary cross-entropy* loss function and *Adam* optimizer are used to optimize the model with a mini-batch size of 128 samples. To train the model, 100 epochs were used to reduce the errors.

**TABLE 3:** Description of datasets used in this research.

Dataset	Instances of class (0)	Instances of class (1)	Size
DR1	998	282	1,280
DS1	27,000	3,000	30,000
DS2	35,000	5,000	40,000
DS3	32,086	3,558	35,644
DS4	40,086	5,558	45,644

**TABLE 4:** Performance analysis of classifiers on dataset DR1 (#features = 44, #rows = 1,280).

Classifier	Parameters	F1-score (%)	Precision (%)	Recall (%)	Accuracy (%)
DNN	4 hidden layers	91.51	92.24	93.13	93.16
KNN	*K* = 3	89.31	88.54	90.94	90.95
RF	Max_depth = 10 # Trees = 100	90.27	89.94	91.86	91.89
DT	Max_depth = 10	89.43	88.96	90.00	90.12
LR		90.42	90.42	90.41	90.55
SVM		90.13	90.13	90.94	90.95

**TABLE 5:** Performance analysis of classifiers on DS1 dataset.

Classifier	Parameters	F1-score (%)	Precision (%)	Recall (%)	Accuracy (%)
DNN	4 hidden layers	95.29	95.25	95.46	95.56
KNN	*K* = 5	93.74	94.68	94.65	94.65
RF	Max_depth = 10 # Trees = 100	88.50	92.39	91.7	91.7
DT	Max_depth = 10	94.66	94.62	94.7	94.7
LR		88.08	89.37	90.93	90.93
SVM		91.96	91.51	91.65	91.97

**TABLE 6:** Performance analysis of classifiers on DS2 dataset.

Classifier	Parameters	F1-score (%)	Precision (%)	Recall (%)	Accuracy (%)
DNN	4 hidden layers	96.96	96.94	96.98	96.99
KNN	*K* = 5	94.43	94.53	94.50	94.50
RF	Max_depth = 10 # Trees = 100	85.11	88.31	86.26	86.22
DT	Max_depth = 10	90.81	90.88	90.76	90.76
LR		90.48	90.50	91.40	91.40
SVM		92.96	93.51	93.65	93.65

**TABLE 7:** Performance analysis of classifiers on DS3 dataset.

Classifier	Parameters	F1-score (%)	Precision (%)	Recall (%)	Accuracy (%)
DNN	4 hidden layers	94.55	94.51	94.73	94.73
KNN	*K* = 5	87.85	87.09	90.79	90.79
RF	Max_depth = 10 # Trees = 100	86.41	82.43	90.79	90.70
DT	Max_depth = 10	89.18	88.59	90.09	90.09
LR		93.38	93.35	93.47	93.47
SVM		93.93	93.94	93.94	93.94

**TABLE 8:** Performance analysis of classifiers on DS4 dataset.

Classifier	Parameters	F1-score (%)	Precision (%)	Recall (%)	Accuracy (%)
DNN	4 hidden layers	95.80	95.80	95.80	95.80
KNN	*K* = 5	78.31	79.10	80.35	80.35
RF	Max_depth = 10 # Trees = 100	68.67	80.62	76.63	76.63
DT	Max_depth = 10	80.15	80.11	80.18	80.18
LR		93.54	93.50	93.85	93.85
SVM		93.16	93.04	93.16	93.16

Tables 5–8 represent the results obtained after the application of different classifiers on datasets DS1, DS2, DS3, and DS4. For KNN, we got the best value for *k* = 5. The max_depth for RF is 10, and the number of trees is 100 and we set the max_depth for DT is 10. For DNN, the architecture has four hidden layers, with 128, 64, 32, 16 nodes in the respective layers. The hidden layer activations functions are ReLu. The output layer has one neuron and Sigmoid activation function. The binary cross-entropy loss function and Adam optimizer are used to optimize the model with a mini-batch size of 128 samples. To train the model, 100 epochs were used to reduce the errors.

**FIGURE 2:**
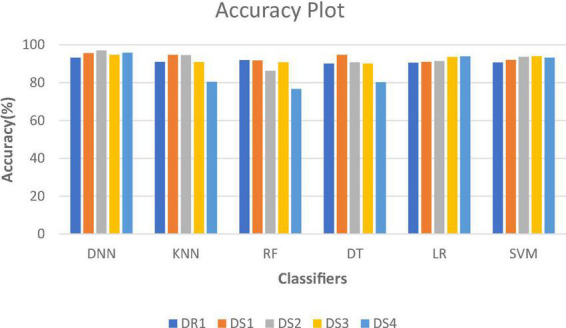
Accuracy plot for different datasets.

Figure 2 shows the comparative analysis of accuracy for all datasets, and it is seen that all the algorithms perform quite well on all datasets. The values for datasets DS1, DS3, and DR1 vary between 91.7 and 95.56%; 90.09 and 94.73%; and 90.12 and 93.16%, respectively. This shows that the patient data can be prepared in such a manner that robust classification models can be built.

A comparison of our work with other research works is presented in Table 9. It can be seen that all the classifiers perform quite well, as compared to other studies as reported in literature. The performance of DNN model is best on all parameters with an accuracy of 96.99%, AUC of 0.984 and recall of 0.9696. The performance of DT and KNN model is only slightly lower than that reported by Abdulkareem et al. (2021), but better than all others. The results of RF are also above 90%.

**TABLE 9:** Comparison of results with other research work.

Study	Dataset used	Classifier used	Accuracy (%)	AUC	F1-score
Mondal et al. (2020)	Hospital Israelita Albert Einstein, Brazil	MLP	93.13	0.96	0.93
Zobai et al. (2021)	Hospital Israelita Albert Einstein, Brazil	SVM, RF	–	0.84	0.72
Jiang et al. (2020)	Wenzhou Central Hospital and Cangnan People’s Hospital, China	SVM	80.00	–	–
Schwab et al. (2020)	Hospital Israelita Albert Einstein, Brazil	XGB	–	0.66	–
BurakAlakus and Turkoglu (2020)	Hospital Israelita Albert Einstein, Brazil	CNNLSTM	92.30	0.90	0.93
Abdulkareem et al. (2021)	Hospital Israelita Albert Einstein, Brazil	SVM	95	0.95	0.94
Turabieh et al. (2021)	Hospital Israelita Albert Einstein, Brazil	CNN	80	–	0.78
Banerjee et al. (2020)	Hospital Israelita Albert Einstein, Brazil	ANN	90	0.95	–
Podder et al. (2021)	Hospital Israelita Albert Einstein, Brazil	XGBoost	92.67	–	0.93
Adem and Kilicarslan (2021)	Hospital Israelita Albert Einstein, Brazil	CNN	92.52	–	–
Our work	Hospital Israelita Albert Einstein, Brazil	**DNN (DS2)**	**96.99**	**0.984**	**0.9696**
		KNN (DS1)	94.65	–	93.74
		RF (DR1)	91.89	–	90.27
		DT (DS1)	94.7	–	94.66

Bold values are highest values.

### Execution time

Table 10 represents the execution time using DNN as for other classifiers the accuracy is less than the DNN classifier so we have measured the time in minutes using DNN.

**TABLE 10:** Execution time of different datasets.

Datasets	Time (minutes) with DNN classifier
DR1	0.15
DS1	4.22
DS2	5.21
DS3	4.23
DS4	5.23

## Discussion

We now present an analysis of best results for the different datasets with respect to the features, size of the dataset and the execution time. The DR1 dataset has 44 features and 1,280 rows and gives the best accuracy of 93.16% for DNN classifier, with an execution time of 0.15 min. The DS1 dataset has 37 features and 30,000 rows and gives the highest accuracy of 95.56% for DNN classifier, with an execution time of 4.22 min. The DS2 dataset has 37 features and 40,000 rows and gives the highest accuracy of 96.99% for DNN classifier, with an execution time of 5.21 min. This is also the overall highest accuracy obtained. The DS3 dataset has 37 features and 35,644 rows and gives the highest accuracy of 94.73% for DNN classifier, with an execution time of 4.23 min. The DS4 dataset has 37 features and 45,644 rows and gives the highest accuracy of 95.80% for DNN classifier, with an execution time of 5.23 min. All our experiments give promising results, thus motivating the researchers to apply feature selection and data augmentation techniques for building machine learning models.

## Conclusion

Real-world data analysis deals with massive, complex data with a several dimensions and volume, and it almost always necessitates some kind of pre-processing. Pre-processing or alteration of this data has an impact on the accuracy of the prediction for which it is utilized and is thus a significant factor to consider. The work presented in this study is to prepare data in such a way that it may be used as input for a later machine learning task of classifying COVID-19 patients, for which data pre-processing, feature selection, and data augmentation were used. This results in creation of five datasets, namely DR1, DS1, DS2, DS3, and DS4. The KNN, DTs, RF, LR, Support vector machine, and DNN classifiers were applied on all the datasets for prediction of COVID-19 disease. Results obtained have been compared with other research work, and it is seen that the proposed DNN model performs the best. It can be concluded that the machine learning techniques may be applied to datasets for efficient classification of diseases. We also believe that with the caution by experts that new virus strains may explode as a pandemic in the future, the proposed model would serve as a base model for transfer learning for future predictive analysis. Further, pandemics like Covid and the resultant social media explosion on do’s and don’ts results in a permanent stress on patients, and careful data screening may be needed, which has also been taken care in the feature selection techniques that we have used. Moreover, we have also shown from our experiments, that, careful data preparation in terms of pre-processing, feature selection and data augmentation is required to get meaningful results. Our future work comprises further adapting our developed models on real life healthcare data so as to provide better and reliable solutions with stress free living.

## Data availability statement

The original contributions presented in this study are included in the article/Supplementary material, further inquiries can be directed to the corresponding author.

## Ethics statement

Ethical review and approval was not required for the study on human participants in accordance with the local legislation and institutional requirements. Written informed consent from the patients/participants or patients/participants legal guardian/next of kin was not required to participate in this study in accordance with the national legislation and the institutional requirements.

## Author contributions

All authors listed have made a substantial, direct, and intellectual contribution to the work, and approved it for publication.
