# Supplementary material for: Improved disease diagnosis system for COVID-19 with data refactoring and handling methods
Source: Front Psychol. 2022 Aug 12;13:951027. doi: 10.3389/fpsyg.2022.951027 (PMC9416861; doi:10.3389/fpsyg.2022.951027)
Supplement: Supplementary file 1 [file Table_1.DOC]

**Appendix A**

| Patient ID | Metapneumovirus | Urine - Hemoglobin |
| --- | --- | --- |
| Patient age quantile | Parainfluenza 2 | Urine - Bile pigments |
| SARS-Cov-2 exam result | Neutrophils | Urine - Ketone Bodies |
| Patient addmited to regular ward (1=yes 0=no) | Urea | Urine - Nitrite |
| Patient addmited to semi-intensive unit (1=yes 0=no) | Proteina C reativa mg/dL | Urine - Density |
| Patient addmited to intensive care unit (1=yes 0=no) | Creatinine | Urine - Urobilinogen |
| Hematocrit | Potassium | Urine - Protein |
| Hemoglobin | Sodium | Urine - Sugar |
| Platelets | Influenza B rapid test | Urine - Leukocytes |
| Mean platelet volume | Influenza A rapid test | Urine - Crystals |
| Red blood Cells | Alanine transaminase | Urine - Red blood cells |
| Lymphocytes | Aspartate transaminase | Urine - Hyaline cylinders |
| Mean corpuscular hemoglobinconcentration\xa0(MCHC) | Gamma-glutamyltransferase\xa0 | Urine - Granular cylinders |
| Leukocytes | Total Bilirubin | Urine - Yeasts |
| Basophils | Direct Bilirubin | Urine - Color |
| Mean corpuscular hemoglobin (MCH) | Indirect Bilirubin | Partial thromboplastin time\xa0(PTT)\xa0 |
| Eosinophils | Alkaline phosphatase | Relationship (Patient/Normal) |
| Mean corpuscular volume (MCV) | Ionized calcium\xa0 | International normalized ratio (INR) |
| Monocytes | Strepto A | Lactic Dehydrogenase |
| Red blood cell distribution width (RDW) | Magnesium | Prothrombin time (PT) Activity |
| Serum Glucose | pCO2 (venous blood gas analysis) | Vitamin B12 |
| Respiratory Syncytial Virus | Hb saturation (venous blood gas analysis) | Creatine phosphokinase\xa0(CPK)\xa0 |
| Influenza A | Base excess (venous blood gas analysis) | Ferritin |
| Influenza B | pO2 (venous blood gas analysis) | Arterial Lactic Acid |
| Parainfluenza 1 | Fio2 (venous blood gas analysis) | Lipase dosage |
| CoronavirusNL63 | Total CO2 (venous blood gas analysis) | D-Dimer |
| Rhinovirus/Enterovirus | pH (venous blood gas analysis) | Albumin |
| Mycoplasma pneumoniae | HCO3 (venous blood gas analysis) | Hb saturation (arterial blood gases) |
| Coronavirus HKU1 | Rods # | pCO2 (arterial blood gas analysis) |
| Parainfluenza 3 | Segmented | Base excess (arterial blood gas analysis) |
| Chlamydophila pneumoniae | Promyelocytes | pH (arterial blood gas analysis) |
| Adenovirus | Metamyelocytes | Total CO2 (arterial blood gas analysis) |
| Parainfluenza 4 | Myelocytes | HCO3 (arterial blood gas analysis) |
| Coronavirus229E | Myeloblasts | pO2 (arterial blood gas analysis) |
| CoronavirusOC43 | Urine - Esterase | Arteiral Fio2 |
| Inf A H1N1 2009 | Urine - Aspect | Phosphor |
| Bordetella pertussis | Urine - pH | ctO2 (arterial blood gas analysis) |
